# Pain Location and Intensity During the First Week Following Coronary Artery Bypass Graft Surgery

**DOI:** 10.5812/aapm.10386

**Published:** 2013-12-26

**Authors:** Ziae Totonchi, Somayeh Seifi, Mitra Chitsazan, Alireza Alizadeh Ghavidel, Farah Baazm, Seyedeh Zahra Faritus

**Affiliations:** 1 Rajaei Cardiovascular, Medical and Research Center, Iran University of Medical Sciences, Tehran, Iran

**Keywords:** Coronary Vessels, Brief Psychiatric Rating Scale, Pain

## Abstract

**Background:**

Despite the advances in pain control following surgery, data on the location and distribution of pain following coronary artery bypass grafting (CABG) are lacking.

**Objectives:**

This study was intended to investigate the location, distribution, and intensity of pain in patients undergoing CABG during their postoperative hospital stay from the operation to the end of the first postoperative week. Factors that could affect pain intensity and distribution were analyzed as well.

**Patients and Methods:**

The present study was conducted on 138 patients who underwent CABG surgery at Rajaei cardiovascular, Medical and Research Center during May and July 2011. Location and intensity of pain were assessed using numeric rating scale (NRS) over time: every six hours after the operation on the first day (T1-T4, respectively), and on two (POD2), three (POD3), and seven days after the operation (POD7).

**Results:**

Among 138 patients assessed in the study, the greatest severity of pain was reported on T2, with the mean severity of 3.4, followed by POD2 with the mean severity of 2.9 (P < 0.01). The location of the surgical incision had the most severity of pain in all patients (P < 0.01). On the site of surgical incision, a negative correlation was seen between the age and the severity of pain on T1 (P = 0.03, r = -0.180). Women experienced more severe pain compared to men at POD7. A significant correlation was seen between the severity of pain on POD7 and body mass index (BMI) (P < 0.01, r = 0.23). In patients who had the longer duration of cardiopulmonary bypass (CBD), the most pain intensity was reported on T1 (P < 0.01, r = 0.18). A significant correlation was seen on the pain intensity on T4 and chest tube drainage (P < 0.01, r = 0.24). The correlation between the pain severity pain and duration of admission in intensive care unit (ICU), was significant on T1 (P < 0.05, r = 0.18), T4 (P < 0.01, r = 0.29), POD2 (P < 0.01, r = 0.35) and POD7 (P < 0.05, r = 0.18).

**Conclusions:**

Following CABG, the most severity of pain was reported at surgical incision on time T2. Pain began to decrease from the third day following the operation. Age, sex and BMI along with operation-related factors such as duration of CBP or chest tube drainage may affect the pain pattern following CABG surgery.

## 1. Background

The World Health Organization in 2002 reported that coronary heart disease causes about 7.2 millions of deaths per year. With increasing incidence of coronary artery diseases, Coronary artery bypass grafting (CABG) has become one of the most common cardiac surgeries performed all over the world (1, 2). Approximately 300,000 patients undergo CABG surgery annually in the United States with an initial hospital cost of around $30,000 for each patient. Despite the advances in pain control following surgery by means of analgesic drugs and nonpharmacological techniques, this is still considered an important problem encountered during postoperative period. Pain following cardiac surgeries can be caused by several factors including: incisions, tissue retraction and dissection during operation, electrocautery and chest tube insertion after the operation. Pain has been reported as one of the most common sources of concern to patients in intensive care unit (ICU) (3-5). Especially after cardiac surgeries (6, 7). It has been suggested that exploring basic mechanisms of postoperative pain may help to optimize the management of acute postoperative, and may lead to develop new treatment strategies (8). Pain specialists believe that the scientific knowledge of pain still needs to be improved (9). Moreover, considering the severity of pain experienced by patients seems to be an important issue to optimize postoperative pain management (10).

A vast majority of research exists regarding the pain following surgeries, but a few studies have focused on cardiac surgery patients (7, 11-14). Moreover, most of these literatures have focused on assessing pain intensity or treatment strategies (15), and only a few data exist regarding the location and distribution of pain after cardiac surgery (16).

## 2. Objectives

This study was intended to investigate the location, distribution, and intensity of pain in patients undergoing CABG during their postoperative hospital stay from the operation to the end of the first postoperative week. Factors that could affect pain intensity were analyzed as well.

## 3. Patients and Methods 

### 3.1. Study Design

This study employed a longitudinal approach to evaluate the pain patterns of patients after CABG surgery over time: every six hours after the operation on the first day (T1-T4, respectively), and on two (POD2), three (POD3), and seven days after the operation (POD-7). 

### 3.2. Primary and Secondary End Points

The primary end point of the present study was to assess the location and intensity of pain in patients undergoing CABG during their postoperative hospital stay from the operation to the end of the first postoperative week. Secondary end points were investigating factors able to affect pain intensity.

### 3.3. Sample and Setting

**Figure 1.**
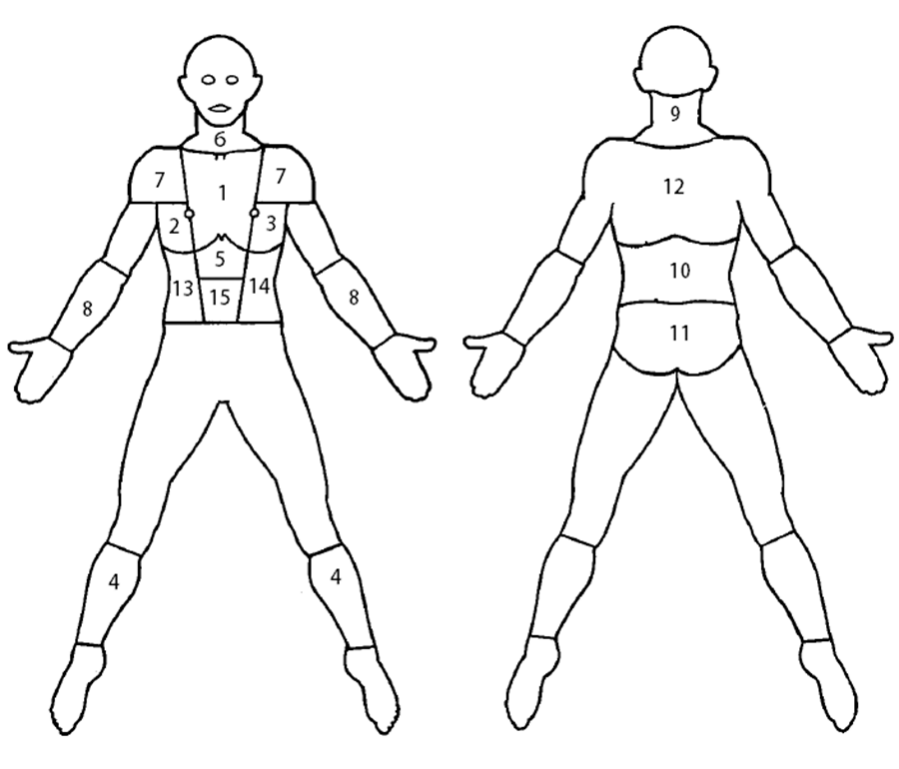
Body Picture with 15 Anatomic Areas

This prospective study was conducted on 138 consecutive patients, American Society of Anesthesiologists (ASA) physical status class II or III, scheduled to undergo CABG with conventional method (sternotomy) in Rajaei Cardiovascular, Medical and Research Center, Tehran, Iran, during May and July 2011. Inclusion criteria were as follows: 1) extubation before the first morning following the operation; 2) ability to seeing, hearing and speaking; 3) staying in hospital during the first week after the operation. Patients undergone surgery emergently, those who needed balloon pump postoperatively, and those undergone the second operation at the same admission were excluded from the study. Patients with a history of opium addiction were also excluded. Before the study, the Numeric Rating Scale (NRS) was introduced to all of the patients. Patients learned how to use the NRS to represent the severity of their pain. To unify described anatomic locations of the pain in all patients a schematic picture of the body was designed (Figure 1). Patients were also instructed to report the location of pain using this picture. In this picture, body has been divided into 15 segments. The surgical incision (sternum) was illustrated as location one (LOC1). 

### 3.4. Data Collection 

Informed written consent was obtained from all patients, and the research project was approved by the ethics committee of Tehran University of Medical Sciences. Premedication included oral lorazepam (1 mg) the night before the operation, and intramuscular morphine (0.1 mg/kg) and oral lorazepam (1 mg) approximately one hour before the operation for all patients. Anesthesia was induced by midazolam (0.1 mg/kg), sufentanil (0.1-0.3 µg/kg), and atracurium (0.5 mg/kg). All patients underwent standard bypass with oxygenation and moderate hypothermia. Anesthesia was maintained using midazolam (1 µg/kg/min), sufentanil (0.005-0.01 µg/kg/min), and atracurium (6 µg/kg/min). Hands were positioned along the trunk on the operation bed, and sternum was opened with wire. Thoracic and mediastinal drains were inserted in rectus abdominis muscle, just under the xiphoid process. Saphenous vein, in the absence of varices, and internal mammillary artery were used for grafting. Patients were monitored during the operation by electrocardiogram, central venous pressure, pulse oximetry, arterial blood gases, and core temperature. Intravenous inotropes or nitroglycerine was used during the operation, as needed. 

Pain was assessed with the numeric rating scale four times including every six hours on the first morning, and on second, third and seventh days after the operation. Based on the NRS, score zero reveals “absence of pain” and score 10 demonstrates “the most severe pain imaginable”. Patients used the schematic picture to localize their pain. During the study severe and intolerable pain was controlled with adequate doses of opiate analgesics or Apotel (intravenous acetaminophen), as required. 

### 3.5. Statistical Analysis

Data were analyzed by Statistical Package for Social Sciences (SPSS) version 16 software (Chicago, IL, The USA). For each of the measured variables or indexes, descriptive values were expressed as the mean ± SD. All data initially were analyzed using the Kolmogorov-Smirnov test to assess the normality. For analysis of quantitative variables, One-Way ANOVA and repeated measurement AVOVA were used. Mean values were compared using t-test, and the chi-squared test or Fisher's test when appropriate. Relationships were assessed using Pearson, Spearman or Kendall tests as appropriate. All P-values were two-tailed, and P < 0.05 was considered statistically significant.

## 4. Results 

One hundred-thirty eight patients including 68 (49.3%) male and 70 (50.7%) female were enrolled in the study. The mean age was 61.43 ± 9.09 years (range, 30 to 85 years). The mean body mass index (BMI) was 27.66 ± 4.48. Table 1 shows the demographic data of the patients. 

**Figure 2.**
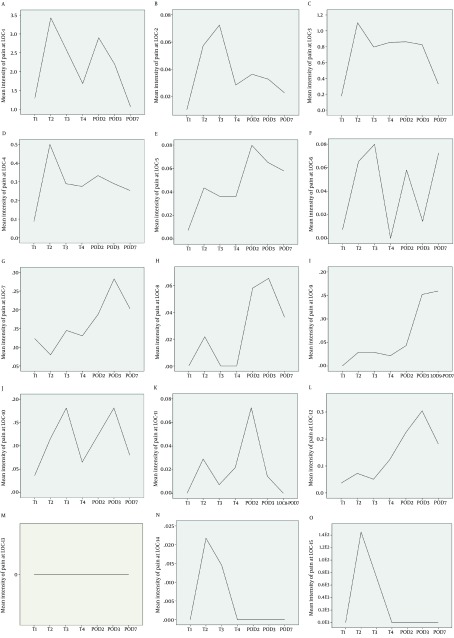
Mean Intensity of Pain at 15 Locations (LOC1-15) During the First Week Following CABG Surgery

**Figure 3.**
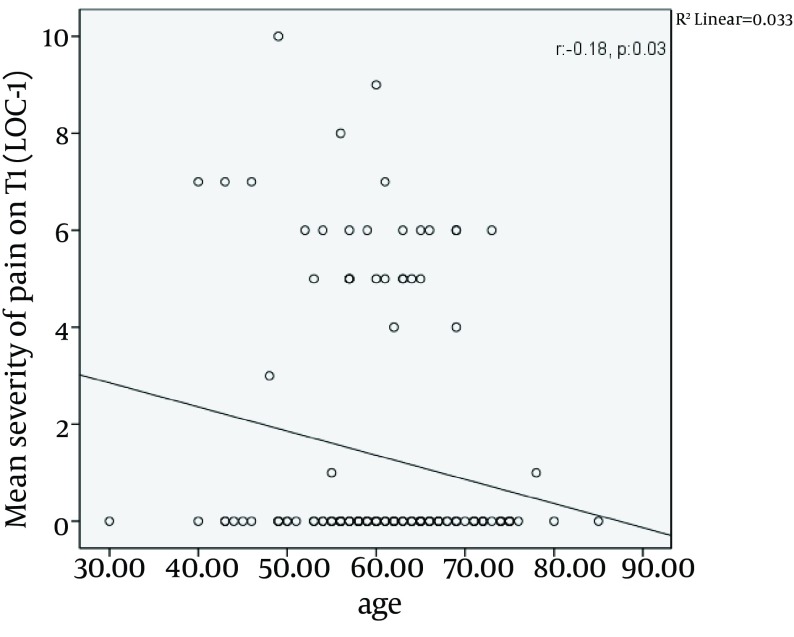
The Mean Severity of Pain on T1 Had a Negative Correlation with Age at the location of surgical incision (LOC1), the mean intensity of pain on T1 had a negative correlation with age.

**Figure 4.**
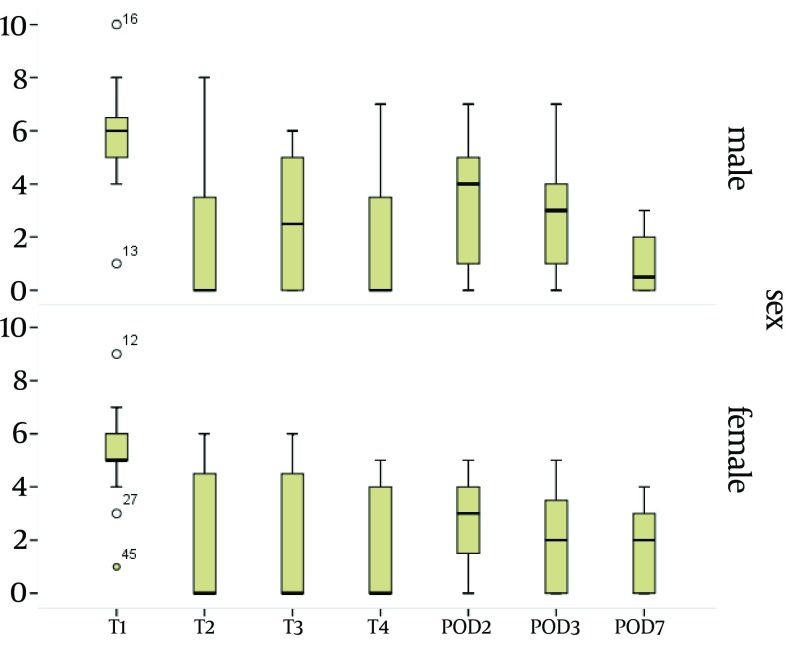
Comparison of the Intensity of Pain on T1 Between Male and Female at Surgical Incision (LOC-1)

Among 138 patients assessed in the study the greatest intensity of pain at the LOC1 was reported on T2, with the mean intensity of 3.4, followed by POD2 with the mean intensity of 2.9 (P < 0.01) (Figure 2). The pain severity at the surgical incision during the study period is demonstrated in Table 2. Analysis of the intensity of pain at the surgical incision over time revealed that there were statistically significant differences between the intensity of pain between T1 and T2 (P < 0.05), between T1 and T3 (P < 0.05), between T1 and POD2 (P < 0.05), and between T1 and POD3 (P < 0.05), while no differences were seen between the intensity of pain between T1 and T4, and between T1 and POD7. During the study, the surgical incision had the most severity of pain in all patients (P < 0.01). On the site of surgical incision, a negative correlation was seen between the age and the pain intensity on T1 (P = 0.03, r = -0.180) (Figure 3). No significant correlation was observed between age and the pain intensity in other locations and at other times. Women experienced more severe pain compared to men at POD7 (Table 3). The differences in the mean intensity of pain between male and female at the surgical incision are shown in Figure 4. On POD7, patients with BMI > 30 complained of the most severe pain among all of the patients (P < 0.01). A significant correlation was seen between the pain intensity on POD7 and BMI (P < 0.01, r = 0.23). In patients who had the longer duration of CBD, the most intensity of pain was reported on T1 (P < 0.01, r = 0.18). When correlation between duration of chest tube drainage and the pain severity was assessed, a significant correlation was seen on T4 (P < 0.01, r = 0.24). In patients whose duration of chest tube drainage was more than three days, the most severe pain was reported on T4 (p < 0.01).

The correlation between the pain severity and the duration of admission in ICU was significant on T1 (P < 0.05, r = 0.18), T4 (p < 0.01, r = 0.29), POD2 (p < 0.01, r = 0.35), and POD7 (p < 0.05, r = 0.18). 

**Table 1.** Demographic Data of Patients Undergoing Coronary Artery Bypass Graft Surgery

	Mean	SD	Maximum	Minimum
**Male/ Female, No.**	68/70			
**Age, y**	61.43	9.09	85	30
**Weight, kg**	72.11	11.23	106	42
**Height, cm**	161.82	8.68	180	142
**BMI^a^, kg/m^2^**	27.66	4.48	41.40	16.40
**AOX^a^time, min**	46.19	19.85	51.50	41.25
**CPB^a^time, min **	86.70	28.46	90.62	76.84
**Operation time, min**	252.76	50.93	260.52	241.54

^a^Abbreviations: AOX, aortic cross-clamping; BMI, body mass index; CPB, cardiopulmonary bypass

**Table 2.** The Pain Severity at Surgical Incision

	Data, Mean ± SD
**T1**	1.29 ± 2.5
**T2**	3.43 ± 2.6
**T3**	2.56 ± 2.51
**T4**	1.68 ± 2.16
**POD2**	2.90 ± 2.07
**POD3**	2.02 ± 1.75
**POD7**	1.06 ± 1.28

**Table 3.** Comparison of the Mean Severity of Pain at Surgical Incision Between Males and Females During the First Week Following Operation

Location	Sex		
	Female, Mean ± SD	Male, Mean ± SD	P value
**T1**	1.2 ± 2.3	1.3 ± 2.6	0.700
**T2**	3.2 ± 2.7	3.6 ± 2.7	0.300
**T3**	2.7 ± 2.6	2.4 ± 2.4	0.600
**T4**	1.5 ± 2.1	1.8 ± 2.1	0.400
**POD2**	3 ± 1.9	2.7 ± 2.1	0.300
**POD3**	2.2 ± 1.8	2.1 ± 1.7	0.500
**POD7**	1.3 ± 1.4	0.76 ± 1	0.008^a^

^a^Statistically significant

## 5. Discussion 

Post operation pain has high incidence among patients undergoing cardiac surgeries. To best of our knowledge, this is the first study which investigated post- CABG patients’ pain patterns in the Iranian population. Although in our study population the pain intensity had alterations during the first week following operation, the location of maximal pain intensity did not change significantly. In all assessed time intervals, the pain was conspicuously more severe in the surgical incision site compared to all other parts of the body. 

In the recent study, pain intensity had fluctuations during the first 2 PODs, and started to decline from POD2. Similar results to ours were found in previous literature (12, 16). Our results reemphasized that the pain intensity diminishes during the first postoperative week; whereas, its distribution stays the same. These results have been also shown in a study by Mueller et al. (16). 

As pain is a subjective sensation, the analysis of the effects of patient`s characteristics, such as age, sex and BMI on the severity of pain seems mandatory. Besides, when we are going to assess the severity of pain, some medical conditions that may affect the intensity of pain include duration of intensive care unit (ICU) admission or duration of insertion of chest tubes. Our study demonstrated that patients with younger age experienced more severe pain at the site of surgical incision on T1 compared to older population. However, there are discrepancies in the previous literature regarding the association between ageing and postoperative pain. Several studies have reported that older patients experience lower pain intensity than younger ones (17-19) while others did not find any association between the pain severity and age (20-22). In the study of Mueller et al. (16) patients younger than 60 had more severe pain on POD2. These impacts of age on the severity of postoperation pain intensity have also been shown in studies by Puntillo et al. (23) and Holl et al. (24). The lower intensity of pain in older population may have been resulted from an erroneous data gathering, due to decreased communication skills in this population in comparison with younger patients. However, the elderly may be more susceptible to lose their autonomic function, which in turn can suppress the pain sensation pathways. Our data emphasized that at the end of the first postoperative week BMI can affect the severity of pain at the surgical incision. In our patient population patients with BMI of more than 30 kg/m^2^ had higher intensity of pain in comparison with those with BMI of less than 30 kg/m^2^. However, Mueller et al. (16) believed that patients with BMI of > 30 kg/m^2^ have more painful areas on POD2. In our investigation the only difference between male and female was detected in the intensity of pain on POD7. One week after the operation women had more intensity of pain at the surgical incision in comparison with the male population. However, there was no general consensus regarding the sex-related differences in the intensity and location of postoperative pain (25, 26). Meehan et al. (12) in a study on 50 patients undergoing cardiac surgery found higher pain intensity in females during the first five PODs following operation. Watt-Watson et al. in a study showed that women had more postoperative chest pain in comparison with men both before and after discharge from hospital (27). 

Our study revealed that the intensity of pain during the first week after cardiac surgery has an association with the duration of ICU admission. However besides pain intensity, a wide range of possibilities would predict the duration of ICU admission in patients undergoing cardiac surgery. 

Our results showed that as the duration of chest tube drainage increases, the mean severity of pain increases, especially on T4. In the study of Paiement et al. 22% of patients who have undergone cardiac surgery believed that the drains were their worst experience during postoperation period (7). This study had shed light on the impacts of chest tubes on postoperative pain. Mueller et al. in a study on 80 patients tried to find the effects of chest tube drainage on the intensity and distribution of postoperative pain (28). Their results showed that in patients who underwent cardiac surgery early chest tube removal results in less pain sensation postoperatively. In our investigation it was shown that more prolonged CPB duration was associated with more intensity of pain on T1. This can be explained by the fact that prolonged CPB duration results in increased release of adrenal stress hormones which in turn can affect the pain sensation justly following the operation (22-24). 

In the present study we excluded patients with a history of chronic opium addiction to ameliorate possible effects of opium on pain perception or tolerability. However, it was shown previously that both addicted and nonaddicted patients undergoing CABG have reported comparable body pain (29). 

The major limitation of our study was its relative small sample size. We also did not analyze the effects of either various modalities for pain management (such as intravenous medications, patient-controlled anesthesia devices, and etc.) or the medications used (opioid versus nonopioid analgesics). All of these factors have been shown to affect the postoperative pain management. 

In conclusion among patients undergoing CABG, the most severity of pain was detected as the site of surgical incision on time T2. Pain began to decrease from the third day following the operation. The location of pain did not change during the first week following CABG. Some of patients ‘characteristics such as age, sex and BMI along with operation-related factors such as duration of CBP or chest tube drainage may affect the pain pattern following CABG surgery. 
